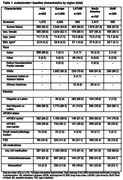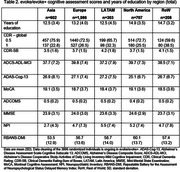# Regional differences in baseline demographic and clinical characteristics from the evoke and evoke+ trials of semaglutide for early Alzheimer’s disease

**DOI:** 10.1002/alz.088793

**Published:** 2025-01-09

**Authors:** Wiesje M. van der Flier, Philip Scheltens, Howard H. Feldman, Oskar Hansson, Mary Sano, Lars Bardtrum, Peter Johannsen, Rose Jeppesen, Charlotte T. Hansen, Teresa Leon, Jeffrey L. Cummings

**Affiliations:** ^1^ Alzheimer Center Amsterdam, Neurology, Vrije Universiteit Amsterdam, Amsterdam UMC, VU University Medical Center, Amsterdam Netherlands; ^2^ EQT Life Sciences Partners, Amsterdam, 1071 DV Amsterdam Netherlands; ^3^ University of California San Diego, La Jolla, CA USA; ^4^ Lund University, Lund Sweden; ^5^ Skåne University Hospital, Malmö, 21428 Skåne Sweden; ^6^ Icahn School of Medicine at Mount Sinai, New York, NY USA; ^7^ James J. Peters VA Medical Center, Bronx, NY USA; ^8^ Novo Nordisk A/S, Søborg, 2860 Søborg Denmark; ^9^ University of Nevada, Las Vegas, NV USA

## Abstract

**Background:**

Differences in patient characteristics across geographical regions may result in heterogeneity in clinical trial populations. evoke (NCT04777396) and evoke+ (NCT04777409) are two phase 3, multinational, randomised trials investigating semaglutide versus placebo in individuals with mild cognitive impairment or mild dementia due to Alzheimer’s disease (AD) (early AD). We present baseline characteristics across the geographical regions in evoke/evoke+.

**Method:**

Baseline data were pooled into five regions (Asia, Europe, Latin America [LATAM], North America and Rest of World [RoW]). Demographic and clinical characteristics were calculated for individual trials (evoke; evoke+), along with pooled data (evoke/evoke+ combined) for each region. Inclusion criteria were the same for both trials; except that evoke+ allowed the inclusion of participants with significant small vessel pathology (>1 lacunar infarct and/or >2 age‐related white matter changes [white matter >20 mm]).

**Result:**

Of the 3,806 individuals with early AD enrolled, there were 1,986, 707, 602, 303 and 208 in Europe, North America, Asia, LATAM and RoW, respectively. Screening failure rates were 54.6‐67.8% across regions and highest in North America. Mean age was 71.7‐73.3 years, and 46.3‐59.1% of participants were female (Table 1). Cognitive scores were similar across regions: mean (standard deviation) Mini‐Mental State Examination score was 23.6 (3.3) to 24.6 (2.9) and Clinical Dementia Rating‐Sum of Boxes score was 3.5 (1.6) to 4.2 (1.8) (Table 2). In evoke+, small vessel pathology was lowest in Europe (2.3%) and highest in Asia (3.8%). Apolipoprotein E4 carrier status was lowest in the LATAM region (51.4%) and highest in the RoW (70.5%), and the proportion of individuals with type 2 diabetes ranged from 11.3‐21.1%. Overall, 51.9‐64.3% of individuals received AD medication, with the highest and lowest use in Asia and North America, respectively.

**Conclusion:**

evoke and evoke+ successfully recruited individuals with early AD from all over the world, ensuring that semaglutide will potentially benefit individuals of various races and ethnicities. Despite this diversity, cognitive scores were largely similar across all geographical regions. Understanding this baseline data will allow for generalisation of the evoke/evoke+ results to worldwide populations with early AD.